# Rhodomycin analogues from *Streptomyces purpurascens*: isolation, characterization and biological activities

**DOI:** 10.1186/2193-1801-2-93

**Published:** 2013-03-09

**Authors:** Sunita Holkar, Deovrat Begde, Nandita Nashikkar, Tukaram Kadam, Avinash Upadhyay

**Affiliations:** Department of Microbiology, School of Life Sciences, Swami Ramanand Teerth Marathwada University (SRTMU), Nanded, 431 401 Maharashtra India; Hislop School of Biotechnology, Hislop College, Temple Road, Civil Lines, Nagpur, Maharashtra 440 001 India

**Keywords:** Streptomyces purpurascens, Taxonomy, Rhodomycin, 16S rRNA, Bioactivity

## Abstract

During a screening program for bioactive natural products, a potential *Streptomyces sp* was isolated from soil. On the basis of biochemical, cultural, physiological and 16S rRNA gene analysis, it was identified as *Streptomyces purpurascens*. The isolate was grown in liquid medium and the crude antibiotic complex was obtained by ethyl acetate extraction. Seven purified fractions were obtained by preparative Thin Layer Chromatography (TLC). Acid hydrolysis of each fraction and subsequent TLC led to the identification of aglycones and sugars indicating these compounds to be Rhodomycin and its analogues. The identity of these compounds was established on the basis of UV-visible and FT-IR spectra and comparison with published data. The compounds were active against Gram-positive bacteria. Compound E, identified as Rhodomycin B, was found to be the most potent compound with an MIC of 2 μg/ml against *Bacillus subtilis*. Compounds A and F identified as α_2_-Rhodomycin II and Obelmycin respectively, and Compound E exhibited an IC_50_ of 8.8 μg/ml against HeLa cell line but no cytotoxicity was found against L929.

**Electronic supplementary material:**

The online version of this article (doi:10.1186/2193-1801-2-93) contains supplementary material, which is available to authorized users.

## Introduction

Anthracyclines, which include rhodomycins, are among the most widely studied natural products of *Streptomyces sp*. over the past quarter century (Vaněk et al. 1977). They attract attention because of their intense color that ranges from yellow and red to purple and blue. They belong to the group of aromatic polyketides where the basic structure is a cyclic polyketide backbone that shares the 7, 8, 9, 10-tetrahydrotetracene-5, 12-quinone structure. The diversity of these secondary metabolites is based on the structural differences in the aglycone and the different sugar residues attached (Additional file 1).

The anthracycline antibiotics have gathered attention of the chemists due to their outstanding antibacterial and antitumor activities (Hortobagyi 1997). Rhodomycin was the first anthracycline compound identified by H. Brockmann in 1963(Brockmann & Bauer 1950). Since then many members of the anthracycline family have been discovered with a broad spectrum of activity. These include daunorubicin, doxorubicin, idarubicin, epirubicin, zorubicin and aclacinomycin A, all of which are obtained from *Streptomyces sp*. The clinical use of these drugs, however has been hampered by a number of undesirable side effects, the most serious being the dose related cardiotoxicity (Jones 1993; Rahman et al. 2007). There is therefore, a great interest in related natural or synthetic compounds having improved therapeutic indices. Many efforts have been made to modify the anthracycline molecule with the objective of developing analogues with a wider spectrum of activity and reduced toxicity (Taatjes et al. 1996;Gianni et al. 2008). Also efforts have been directed towards obtaining hybrid anthracyclines with less toxicity. However, the complexity of the structure and biosynthetic pathway of anthracyclines have posed difficulties in these efforts (Jansson et al. 2003).

Soil is a natural reservoir for microorganisms, particularly the spore forming *Streptomyces* and their antimicrobial products. It is a known fact that *Streptomyces* are an unsurpassed source of bioactive materials. Although soils have been extensively screened by the pharmaceutical industry for about 50 years, only a small fraction of the surface of the globe has been sampled, and only a small fraction of *Streptomyces* taxa has been discovered. There is a strong circumstantial evidence that the discovery of previously unknown natural products occurs when novel organisms are examined in either established or new pharmacological screening programs (Takahashi & Omura 2003). There are probably many unexplored anthracycline producing *Streptomyces* in soil which are yet to be discovered and may have an answer to this glaring problem.

With this intention, a *Streptomyces sp*. was isolated from soil and investigated for several bioactivities. It has been identified as *Streptomyces purpurascens* and deposited in the Microbial Type Culture Collection (MTCC), Chandigarh, India (http://mtcc.imtech.res.in/) with the accession number MTCC 8547. The red colored ethyl acetate cell extract of the isolate showed a high anti-microbial activity and the TLC delivered several red and orange zones. In the present study we report isolation, taxonomy of the isolate, its antimicrobial and anticancer activities, partial purification and characterization of the anthracyclines produced.

## Material and methods

### Isolation of the microorganism

In a systematic screening program for isolation of bioactive *Streptomyces*, soil samples from different locations in and around Nagpur, Maharashtra, India, were collected and stored at -20°C. The soil samples were pretreated by heating at 60°C for 10 minutes. 1 g each of the treated soil sample was serially diluted at 1:10 and 1:100 in sterile distilled water and plated on Starch-casein (SC) agar plates (Thakur et al. 2007). The SC agar plates were incubated for 14 days at 30°C and the resulting colonies were subcultured and maintained on Yeast extract-malt extract (YEME) agar (Ouhdouch et al. 2001;Smaoui et al. 2012).

Unless otherwise mentioned, all the chemicals and solvents were purchased from Merck and culture media from Hi-media laboratories, Mumbai.

### Identification of the isolate

The morphological and cultural characteristics of the isolate were assessed in accordance with the method described by Shirling and Gottlieb (1966); Thakur et al. (2007)^,^. Cultural characteristics were observed on YEME agar (ISP2), Oatmeal agar (ISP3), Inorganic salt Starch agar (ISP4), and Glycerol Asparagine agar (ISP5) after 14 days of culturing at 28°C. The morphological characteristics were assessed *via* light microscopy of 7-day old cultures grown on ISP4. Color of aerial mycelium was determined from mature, sporulating aerial mycelia of the *Streptomyces* colonies on SC agar media (Pridham et al. 1958).

The presence of soluble pigments and the melanoid pigment was investigated on (ISP 2) and peptone yeast extract iron agar (ISP 6).

The diagnostic isomers of diaminopimelic acid (DAP) and whole-organism sugars of the isolate were analyzed by the procedure developed by Becker *et al.*(1965) Utilization of various carbon and nitrogen sources was examined. Each source was added at a final concentration of 1% (w/v) and 0.1% ( w/v) respectively. Degradation of starch was determined according to Gordon *et* al. (1974). Growth in the presence of sodium chloride was determined according to Tresner *et al.* (1971) and temperature and pH requirements were assessed on ISP 2 medium.

### Molecular characterization of the isolate

A confirmatory taxonomic identification was done by the nucleotide sequencing of the 16S rRNA gene. Genomic DNA extraction, amplification and sequencing of the 16S rRNA gene were performed as described earlier (Mayilraj et al. 2006). The 16S rRNA gene was amplified with primers 8-27f (5′-AGAGTTTGATCCTGGCTCAG-3') and 1500r (5′AGAAAGGAGGTGATCCAGCCA-3′). The amplified DNA fragment was separated on 1% agarose gel, eluted from the gel and purified using QIAquick gel extraction kit (Qiagen). The purified PCR product was sequenced with 27f, 519r (5-GWATTACCGCGGCKGCTG-3), 1087r (5-CTCGTTGCGGGACTTAACCC-3), 530f (5-TTCGTGCCAGCAGCCGCGG-3), 945f (5-GGGCCCGCACAAGCGGTGG-3) and 1492r respectively (*Escherichia coli* numbering system). The rDNA sequence was determined by the dideoxy chain-termination method using the Big-Dye terminator kit using ABI 310 Genetic Analyzer (Applied Biosystems, USA). Almost complete sequence (1458 bp) of 16S rRNA of strain MTCC 8547 was determined and was compared with those of other closely related taxa retrieved from the GenBank database. A phylogenetic tree was constructed by Neighbour-Joining plot (Perrière & Gouy 1996). A sequence similarity search was done using GenBank BLASTN (Altschul et al. 1997). Sequences of closely related taxa were retrieved; aligned using Clustal X programme (Thompson et al. 1997) and the alignment was manually corrected. For the Neighbour-joining analysis (Saitou & Nei 1987), the distances between the sequences were calculated using Kimura’s two-parameter model (Kimura 1980). Bootstrap analysis was performed to assess the confidence limits of the branching (Felsenstein 1985).

### Growth and antibiotic production studies

Antibiotic production was studied in Potato dextrose broth (PDB) and antibiotic production medium, GS medium containing glucose 10 g, soyabean meal 10 g, NaCl 10 g and CaCO_3_ 1 g in 1 litre of water (Singh et al. 2009). The pH of PDB was adjusted to 6.8 and that of GS to 7 before sterilization. Seed culture was prepared in 100 ml Erlenmeyer flasks containing 25 ml GS medium by inoculating a loopful from a slant culture and incubating at 28°C in a shaker incubator at 150 rpm for 48 hours. Antibiotic production was observed using the same medium by inoculating 200 ml medium with 2.5% (v/v) of seed culture and cultivating under identical incubation conditions for 8 days.

### Effect of carbon and nitrogen

The effect of various carbon and nitrogen sources was studied on growth as well as antibiotic production. Glucose soyabean meal medium was used as a standard medium. Carbon sources like starch, cellobiose, galactose, mannose, raffinose, rhamnose, lactose, maltose, fructose, sucrose, glycerol and xylose and nitrogen sources like casein, peptone, yeast extract, tryptone and jack bean meal were added at 1% concentration into the standard media with appropriate controls. After 10 days, each flask was harvested, the fermented broth as well as the cells were extracted with ethyl acetate, the extracts were dried in vacuum, resuspended in methanol to get a clear solution and tested for antibiotic activity against *Bacillus subtilis*. Growth was assessed by measuring cell mass.

### Isolation and purification of antimicrobial products

The mycelial cake obtained from 200 ml broth was extracted with 100 ml acetone (Saito et al. 1995) and the spent broth (200 ml) was extracted with 100 ml chloroform. The acetone extract was concentrated to one-fourth original volume and then reextracted with 25 ml chloroform. The resulting chloroform extracts were concentrated and then excess of 60-80°C petroleum ether was added to obtain around 150 mg of crude antibiotic complex (Eckardt et al. 1985).

The resulting dry broth and cell extracts were resuspended in a small volume of methanol and were tested for their antibacterial activity against *B. subtilis* by agar well diffusion method, but there was hardly any appreciable activity in the broth extract and hence it was not pursued further. All the other studies were carried out with only the cell extract. Preparative chromatography with Silica gel plate 60 F 254 (Merck, Germany, Cat # 1.05554.0007) was used for the partial purification of antimicrobial products. The crude extract was spotted and developed in the solvent system Chloroform: methanol: 25% aqueous NH_3_ (85:14:1) (Johdo et al. 1991a; Tobe et al. 1982). The developed plates were air dried overnight to remove all traces of solvents. Seven well separated bands were detected by observations of the color of the bands, which were orange to red. The TLC was repeated several times and the mean R*f* of the bands was calculated. The fractions were physically separated from each other by scraping the bands from the plates, extracting with methanol, concentrating the extracts and again subjecting each concentrate to TLC using the same solvent system, thereby obtaining each fraction in substantially pure form.

### Spectral studies

The UV-visible absorption spectra (190 nm-1100 nm) of the TLC purified fractions were determined by using a double beam bio-spectrophotometer, (BL-198, Elico Ltd.). Futhermore, FT-IR spectrum of each active extract was detected using Shimadzu IR-470 plus. The spectra were scanned in the 400 to 4000 cm^-1^ range and plotted as intensity versus wave number (Augustine et al. 2005).

### Qualitative determination of aglycone and sugar residues by TLC

To an aliquot of the purified fraction, 0.5 ml of 0.1 N HCl was added and was heated at 85°C for 30 minutes in a water bath. The pigment aglycone thus obtained was extracted with CHC1_3_. The CHCl_3_ layer was evaporated to dryness and the pigment residue was chromatographed on a Silica gel plate 60 F 254 (Merck, Germany, Cat # 1.05554.0007) using CHCI_3_: MeOH (15 : 1) mixture for chromatographic separation. The reference aglycones ε-rhodomycinone (RMN) (red), ε-isorhodomycinone (isoRMN) (purple), α-citromycinone (CTN) (yellow), α2-RMN (orange), βRMN (red), β-isoRMN (purple) and γ-isoRMN (purple) are documented to have R*f* values of 0.79, 0.77, 0.29, 0.27, 0.45, 0.43 and 0.59, respectively (Johdo et al. 1991a). The aglycones were also determined by spot colors on the TLC plate.

Alternatively, the aqueous layer containing sugar components was neutralized by adding silver carbonate with a small amount of charcoal and centrifuged. The supernatant fluid was concentrated *in vacuo* and chromatographed on TLC Silica gel 60 plate F254 (Merck, Germany, Cat # 1.05554.0007) using a BuOH- acetic acid - H_2_O (4 : 1 : 1) solvent system. The sugar spots were detected with p-anisaldehyde - H_2_SO_4_ (each 5%) in 90%EtOH spray reagent followed by heating at 90° (Johdo et al. 1991a).

### Bioactivity of the fractions

Each crude extract and purified fractions were tested against *Bacillus subtilis, Staphylococcus aureus, Escherichia coli and Pseudomonas aeruginosa* using agar well diffusion technique. 15 μl of 1 mg/ml stock extracts were used for the tests. The diameter of the inhibition zones was determined after 24 hours of incubation at 37°C. MIC of the bioactive fractions was determined using broth dilution technique (Wiegand et al. 2008).

## MTT-based cytotoxicity assay

The cytotoxicity of bioactive fractions on established cell lines like HeLa and L929 was determined *in vitro* as described by Mosmann (1983); Begde et al. (2010). The cell lines were purchased from NCCS, Pune, India. Freshly passaged HeLa cells were centrifuged and washed with HBSS. The cell count was adjusted to 2*.*5 × 10^5^ cells/ml by suspending the cells in fresh DMEM with 10% FBS (Invitrogen). This cell suspension was then transferred to a 96-well TC plate for the assay and the cells were allowed to grow for 24 hrs at 37°C. The spent medium was discarded and the adherent cells were exposed to a concentration gradient of each purified fraction supplemented in the medium to a final concentration of 2.5, 5, 10, 15, 20, 25, 30 and 35 μM respectively. The plate was incubated at 37°C in a humidified environment with 5% CO_2_ in air for 48 h. After incubation the cells were washed and resuspended in fresh medium containing MTT (Himedia) and were further incubated for 3 hrs. The MTT formazan produced by the viable cells was extracted in DMSO (BIOGENE Reagents Inc., CA, USA) and quantified at 570 nm in the Bio-Rad ELISA plate reader.

## Results

During a screening program for natural bioactive products, several microorganisms were isolated and their antimicrobial activity was studied. One of the cultures showing antibacterial activity was found to be promising and studied further.

### Taxonomy of the producer organism

The morphological, physiological and cultural characteristics indicated that the soil isolate belonged to the genus *Streptomyces. Streptomyces* belong to the family Streptomycetaceae of the order Actinomycetales (Shirling & Gottlieb 1966). The physiological characteristics and the cultural characteristics of the isolate are summarized in the Tables 1 and 2 respectively.

**Table 1:** **Physiological/Biochemical characteristics of*Streptomyces purpurascens***

Characteristics	***S.purpurascens***
Temperature range of growth	25-42°C
Urea hydrolysis	-
Starch hydrolysis	+
Caesin hydrolysis	-
Gelatin hydrolysis	+
Nitrate reduction	-
H_2_S production	-
Citrate utilization	+
Catalase	+
Oxidase	-
Arabinose	+
Dextrose	+
Galactose	+
Fructose	+
Inositol	+
Raffinose	+
Rhamnose	+
Sorbitol	-
Salicin	-
Sucrose	+
Xylose	-
Mannitol	+
Lactose	-

+: Positve; -: negative.

The table details the biochemical properties associated with the isolate providing the biochemical guidelines towards identity of the organism.

**Table 2:** **Cultural characteristics of*S. purpurascens*MTCC 8547**

Media	Color of mycelium	Spores	Reverse side	Pigment	
ISP1	Orange	White +	Orange	Orange	
ISP2	Orange	White +++	Orange	Orange diffusible	
ISP3	Orange	White +	Orange	Orange diffusible	
ISP4	Orange	White +++	Orange	Orange diffusible	
ISP5	Violet	White +++	Violet	Violet	
ISP6	Yellow	-	Yellow	Brown	
ISP7	Pink	-	Pink	No	
PDA	Orange	White+++	Orange	Orange	
SDA	Orange	White+++	Orange	Reddish orange	

**Figure 1:**
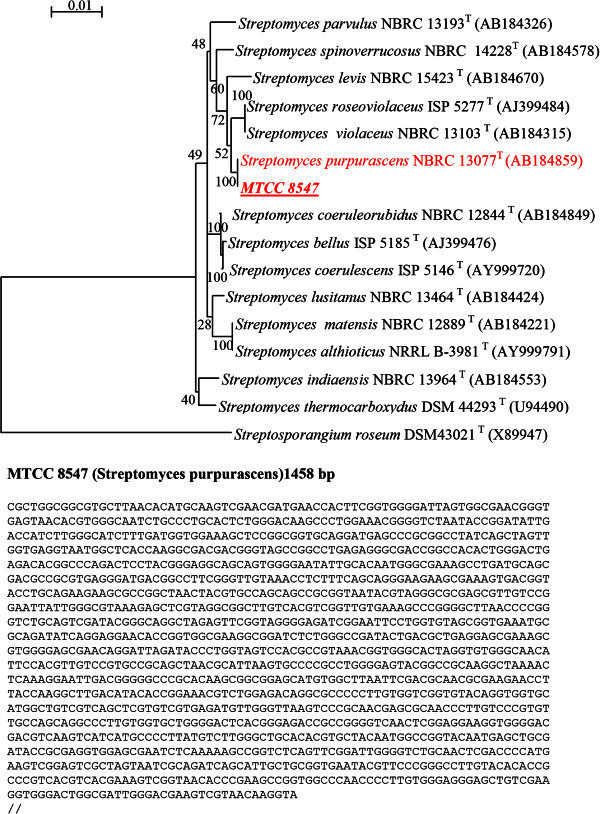
**Neighbour-joining tree based on 16S rRNA (1458 bases) sequences, showing the phylogenetic relationship between strain MTCC 8547 and other phylogenetic neighbours.***Streptosporangium roseum* DSM 43021 ^T^ (X89947) was used as out group. Bootstrap values expressed as percentage of 100 replications. Bar represents 1% sequence variation. Below is 1458 bp 16S rRNA gene sequence determined by the dideoxy chain-termination method using the Big-Dye terminator kit. This sequence was used to perform the sequence similarity search using GeneBank BLASTN based on which the phylogenetic tree was constructed by NJ plot.

The comparison of the partial sequences (1458 bp) of 16S rRNA of the isolate with those found in databases was done by BLASTN analysis. Result showed that query sequence of the isolate was best pair-wise aligned with 16S rRNA partial gene sequence of *Streptomyces purpurascens* strain: KN-6 (AB231806.1, score value 2751 bits) with similar sequence homology and identity of 100%. The N-J phylogenetic tree of the 16S rDNA sequence data confirmed that the isolate was most closely related to *S. purpurascens* (Figure 1)*.*

Out of 1458 16S rRNA nucleotides of the isolate all were identical with those of *S. purpurascens NBRC 13077*, indicating that the sequence similarity of the 16S rRNA of the two bacteria was 100%. The culture has been deposited at Microbial Type Culture Collection, Institute of Microbial Technology, Chandigarh, India (MTCC; http://mtcc.imtech.res.in/) as MTCC 8547.

### Effect of carbon and nitrogen sources

Out of all the carbon sources tested for antibiotic production, Starch was found to be the best followed by cellobiose, raffinose, mannose and fructose. Of the nitrogen sources tested for antibiotic production, Yeast extract was the best followed by peptone and casein (Additional file 2: Figure S1).

### Physicochemical properties

The purified fractions were named as **A, B, C, D, E, F and G**. The stock solutions were preserved at -20°C.

**Figure 2:**
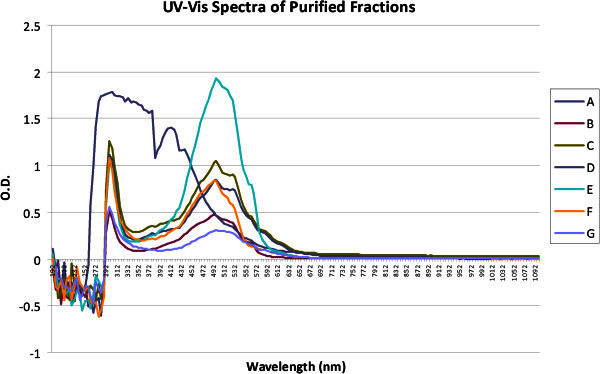
**The UV visible absorption spectra of all the purified fractions, except D, exhibited characteristic peaks of anthracyclines in the range of 297 nm, 492- 497 nm, 522- 526 nm and 557 -562 nm.** Absorption spectra for all the fractions were recorded in methanol, against methanol blank.

The UV visible light absorption spectra of the purified fractions in methanol, against methanol blank exhibited characteristic peaks of anthracyclines in the range of 297 nm, 492- 497, 522- 526 and 557 -562 (except D) (Figure 2).

**Figure 3:**
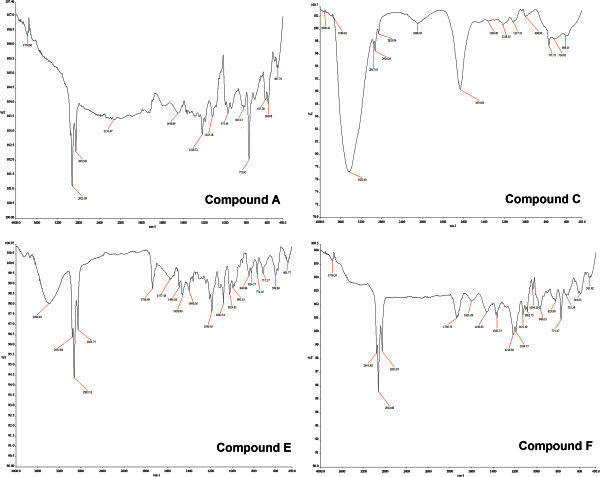
**FT-IR spectrum of some major purified compounds.** The purified and dried compounds were ground in KBr and absorbances were recorded in the range 400-4000 cm^-1^ to generate the FT-IR spectra. The FT-IR Spectra of four major compounds **A**, **C**, **E** and **F** are shown here. All purified fractions consistently indicated the presence of hydroxyl group (3400-3300 cm^-1^) a ketonic group or ester carbonyl (1740 cm^-1^) and a hydrogen bonded carbonyl (1600 cm^-1^) which are characteristics of anthracyclines. For more detailed FT-IR peak positions refer Table 3.

The FT-IR spectra (Figure 3) of all purified fractions indicated the presence of hydroxyl group (3400-3300 cm^-1^) a ketonic group or ester carbonyl (1740 cm^-1^) and a hydrogen bonded carbonyl (1600 cm^-1^) which is characteristic of anthracyclines (Oki 1979). The properties of all fractions with their corresponding IR peaks are summarized in Table 3.

**Table 3:** **Tabulation of the physical and chemical properties of the compounds purified by preparative Thin Layer Chromatography**

Properties	A	B	C	D	E	F	G
**Color**	Red	Orange	Reddish Orange	Yellowish Orange	Orange	Pink	Violet
**Predicted Molecular formula**	ND	ND	C_42_H_51_NO_16_	C_42_H_53_NO_15_	C_36_H_48_N_2_O_12_	C_40_H_53_NO_15_	C_36_H_48_N_2_O_12_
**Melting point (˚C)**	ND	184-190	175-178	151-153	185-190	160-163	134-137
**UV λ**_**max**_**(nm)**	237(2.014),	297(0.508),	237(1.963),	287(1.751),	297(1.023),	297(1.072),	297(0.552),
	257(2.11),	463(sh),	257(sh),	317 (1.74),	497(1.923),	492(0.834),	377(0.101),
	284(1.957),	492(0.472),	495(0.483),	375 (1.59),	517(sh),	527(sh)	497(0.304)
	492(0.614),	526(sh),	522(0.415),	412 (1.401)	561(0.792)		
	522(0.635),	562(sh)	562(sh)	437 (1.172)			
	557(0.461)						
**IR**_**max**_**(KBr) (cm**^**-1**^**)**	3780,	3763,	3760, 3432,	ND	3394, 2922,	3780, 2924,	ND
	2924,	2922,	2924,		1736,	1736,	
	2131,	1723,	1634, 1363,		1577,1490,	1600, 1459,	
	1450, 1218, 1120,971, 823	1570, 1458, 1192,1044, 827	1218, 1117, 998		1458, 1190,1024, 825	1363, 1189, 1034, 823	
**Rf value**^**a**^	0.88	0.80	0.79	0.68	0.62	0.48	0.17

^a^ the values were obtained on silica gel TLC, CHCl_3_: MeOH: 25% aqNH_3_ (85:14:1); ND- Not Determined.

The table provides details about the UV–vis and FT-IR absorption peaks of respective purified compounds necessary for their identification.

### Qualitative determination of aglycone and sugars by TLC

On acid hydrolysis, followed by TLC analysis, the purified fractions separated into red, orange and purple aglycones and corresponding sugar components. Except **D**, whose aglycone was found to be aklavinone, the aglycones of all the fractions were found to be rhodomycinone (RMN). Fractions **A** and **G** gave α2-RMN, **B** and **E** gave β-RMN, **C** gave ε-RMN and **F** gave γ isoRMN on hydrolysis. These aglycones were peri-hydroxy quinones was established by the color change of the red- orange bands to violet on staining with 0.5% ethanolic Magnesium acetate (Romanova et al. 1977). The sugars of all the fractions were found to be L-Rhodosamine and 2-deoxy-L- fucose by direct comparison with standard sugars on TLC. The details of the aglycones and sugars are summarized in Table 4.

**Table 4:** **A detailed description of sugar and aglycone substituent groups of all the isolated anthracyclines obtained from*S. purpurascens***

Fraction	Aglycones		Sugars			Compound predicted
	Rf^a^detected	Aglycone	Rf^b^	Color	Sugar detected	
A	0.27	α_2_ –RMN	0.56	grayish blue	2-deoxy-L- fucose	α_2-_Rhodomycin II
B	0.45	β RMN	0.56	grayish blue	L-Rhodosamine	Rhodomycin
					2-deoxy-L- fucose	
C	0.79	ε RMN	0.56	grayish blue	2-deoxy-L- fucose	Epelmycin B
D	0.67	Aklavinone	0.12	sky blue	L-Rhodosamine	Aclacinomycin A
			0.56	grayish blue	2-deoxy-L- fucose	
E	0.45	β-RMN	0.12	sky blue	L-Rhodosamine	Rhodomycin A or B
			0.56	grayish blue	2-deoxy-L- fucose	
F	0.59	γ-iso RMN	0.12	sky blue	L-Rhodosamine	Obelmycin
			0.56	grayish blue	2-deoxy-L- fucose	
G	0.27	α_2_ –RMN	0.12	sky blue	L-Rhodosamine	Alldimycin B
			0.56	grayish blue	2-deoxy-L- fucose	

a: CHCl_3_ : MeOH (15:1), b: Butanol: acetic acid: water (4:1:1).

Identification of the sugars and aglycones were done by comparing their R*f* with that of the standards under similar TLC solvent conditions.

### Identification of compounds

**Compound A** appeared identical to α_2_-Rhodomycinone glycoside, probably α_2_-Rhodomycin II, which has been previously reported for *S. purpurascens*. **Compound B** was found to contain β-RMN as the aglycone and L-Rhodosamine as the sugar, which indicated that it may be a Rhodomycin. The R*f* and the UV–vis spectra of the aglycone of **B** matched that of β- RMN. Also the UV–vis spectra of **B** was found to be similar to that of S-583 A-II (Shoji et al. 1968), which is reported to be a Rhodomycin B. Therefore it was considered that **B** is similar to Rhodomycin B. **Compound C** on acid hydrolysis yielded ε-rhodomycinone (RMN) and L-Rhodosamine and deoxy fucose as the sugars. Hence it was suspected to be a ε-rhodomycinone glycoside. Johdo *et. al.,* have named such ε-rhodomycinone glycosides from *S. violaceus* as Epelmycins (Johdo et al. 1991b). The UV spectrum of **C** was found to match that of Epelmycin C with prominent peaks at 237 nm, 495 nm and 522 nm. Also the R*f* of purified **C** on silica gel TLC in solvent system CHCl_3_: MeOH (8:1) was found to be 0.22 which coincided to the R*f* of Epelmycin C in the same solvent system as reported by Johdo *et. al*. The FT-IR spectra (Figure 3) of **C** indicated the presence of an ester carbonyl (1730 cm^-1^) and a hydrogen bonded carbonyl (1600 cm^-1^) group which are characteristic of epelmycin anthracyclines (Johdo et al. 1991b). Also the absence of non-chelated carbonyl absorption (1670 cm^-1^) gave an indication that the aglycone could be ε-RMN rather than aklavinone (Doyle et al. 1979). Therefore this Rhodosaminosyl-decyfucosyl ε-rhodomycinone could be an Epelmycin as reported by Johdo *et. al.,* for *Streptomyces violaceus*. **Compound D** was yellow in color showing similarity to Aclacinomycin B. On hydrolysis, it yielded aklavinone and the sugars, Rhodosamine and deoxyfucose. **D** gave an R*f* of 0.68 in CHCl_3_:MeOH:25% aqNH_3_ which is reported for aclacinomycins in the same solvent system. Its UV–vis spectra appeared similar to that of aclcinomycin B with an absorption maxima at 437 nm. Also the aglycone of **D** gave a single yellow band with an R*f* of 0.48 which coincided to the R*f* of standard alkavinone (Connors et al. 1990) under similar conditions. It also exhibited a violet fluorescence which is reported for aklavinone (Batel et al. 1990).

**Compound E** on acid hydrolysis yielded β-RMN and the sugar L-Rhodosamine and even its FT-IR peaks (Table 3 & Figure 3) appeared similar to rhodomycin. Thus it resembled rhodomycin which is reported for *S. purpurascens.* The band of **compound F** on TLC was purple in color which on acid hydrolysis yielded the aglycone γ-iso RMN giving an indication that it may be similar to Obelmycin. The sugars detected in **F** were L-Rhodosamine and deoxyfucose. The fact that **F** contains γ-iso RMN and L-Rhodosamine and deoxyfucose led us to believe that **F** could be Obelmycin F. The belief was further strengthened by both having the same R*f* 0.48 in CHCl_3_:MeOH:NH_3_ (80:10:1). Furthermore, the UV-visible spectra of **F** and Obelmycin F showed coinciding peaks at 297 nm, 492 nm and 527 nm. And even the FT-IR spectra of **F** indicated the presence of hydrogen bonded carbonyl (1600 cm^-1^) which is characteristic of Obelmycins. But Obelmycin F is reported to contain L-Rhodinose along with L-Rhodosamine and 2-deoxy fucose. However, **F** did not show the presence of L-Rhodinose. **F** therefore was concluded to be similar to L-Rhodosaminosyl deoxyfucosyl γ-iso rhodomycinone. **Compound G** on acid hydrolysis yielded α2 rhodomycinone, hinting that it may be a α_2_-RMN glycoside, which have been designated as Alldimycins by Johdo *et. al* in *S. violaceus*. The R*f* of **G** in CHCl_3_:MeOH:NH_3_ was 0.17 which exactly matched that of Alldimycin B (Johdo et al. 1991b). Also the UV–vis spectra showed a prominent peak at 497 nm similar to Alldimycins. Alldimycins have been reported to have only Rhodosamine as the sugar (Johdo et al. 1991b) whereas the hydrolysis product of **G** also yielded a spot for deoxy fucose on the chromatogram. Therefore, **G** perhaps was a new compound, resembling L- rhodosaminosyl, deoxyfucosyl α_2_-rhodomycinone which needs further confirmation.

### Bioactivity of purified compounds

**Figure 4:**
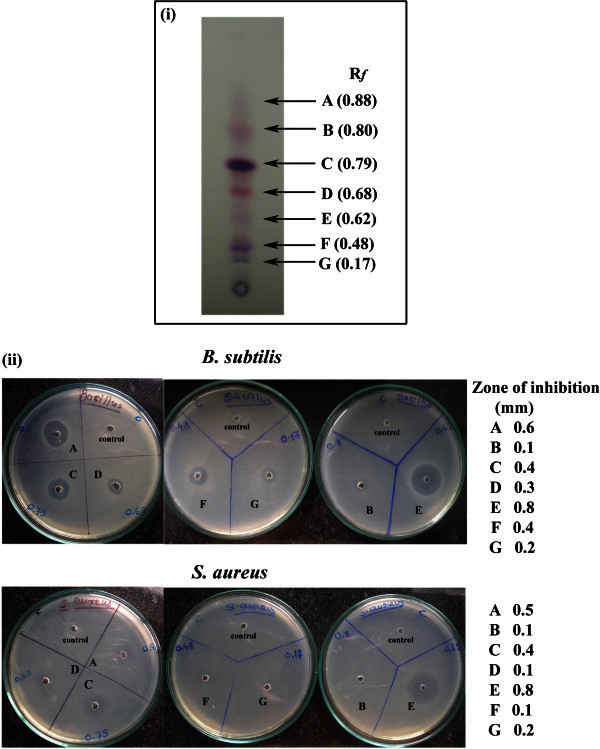
**(i) Preparative TLC- For the partial purification of antimicrobial products preparative thin layer chromatography was performed.** The crude extract got resolved into seven well separated colored bands when developed in the Chloroform: Methanol: 25% aqueous NH_3_ (85:14:1) solvent system. According to the R*f* value, each fraction was named from A to D. **(ii)** Antimicrobial activity of the isolated fractions against *B. subtilis* and *S. aureus* determined by agar well diffusion assay. Zone of inhibition associated with each fraction against respective test organisms is depicted.

Antibacterial and antifungal activities were semi-quantatively determined using agar well diffusion method where, **Compound E** was found to have the highest activity against *B. subtilis* and *S. aureus* followed by compound **A**. Compounds **C** and **F** demonstrated similar activities whereas compounds **D**, **G** and **B** showed very weak activity (Figure 4). All the fractions failed to give any activity against Gram negative bacteria and *Candida albicans.*

The MIC of compounds **E**, **A** and **F** was determined against *B. subtilis* by broth dilution method. The compound **E** was found to be very active with an MIC of 2 μg/ml whereas compounds **A** and **F** were comparatively less active with an MIC greater than 20 μg/ml.

Furthermore, the compounds **E**, **A** and **F** were found to be active against human cervical carcinoma HeLa cells with an IC_50_ of for E ~15 μM. However, in the tested range, these compounds did not demonstrate any appreciable cytotoxicity against mouse subcutaneous fibroblasts, L929 cells.

## Discussion

In our screening program for anthracycline producers from soil, a *Streptomyces sp.* was isolated from soil. The isolate was identified as *Streptomyces purpurascens* by 16S rRNA gene sequencing and deposited in Microbial Type Culture Collection (MTCC), Chandigarh, India (http://mtcc.imtech.res.in/) with the accession number MTCC 8547. The isolate was found to produce rhodomycins which are reported for *S. purpurascens*. Apart from this, some rhodomycin analogues which were not reported for *S. purpurascens* before were also obtained. In the present study at least one new rhodomycin analogue, was obtained from this isolate.

The isolate when grown in liquid culture produced red colored pigments extracellularly. Preliminary testing using the crude cell extract of the isolate showed it to be antibacterial against Gram positive bacteria. However, the crude extracts were not found to be active against Gram negative bacteria nor were they antifungal.

Studies on optimization of the growth medium indicated Starch as the best carbon source for growth as well as antibiotic production whereas yeast extract and peptone served as the best nitrogen source for antibiotic production and for growth respectively (For more details refer to Additional files). Therefore for all the further antibiotic production procedures the Starch-Yeast extract medium was preferred. Solvent extraction of the spent broth and subsequent purification using preparative TLC helped in obtaining the purified compounds. Seven purified compounds thus obtained were named **A, B, C, D, E, F** and **G.**

The UV visible spectra of all the fractions showed a peak in the 230-290 nm region indicating the presence of an aromatic ring in the structure. Also the FT-IR spectra of the compounds indicated the presence of hydroxyl groups, ketonic groups and hydrogen- bonded carbonyl groups which are characteristic of anthracyclines. Hence it was speculated that the isolate was an anthracycline producer. Identification of our isolate as *S. purpurascens* further strengthened the supposition since it is well known to produce anthracyclines belonging to the rhodomycin group (Brockmann & Bauer 1950). On acid hydrolysis each compound yielded an aglycone and deoxy- sugars. The identity of the aglycone and sugars (by TLC) confirmed that these compounds might be rhodomycin and/or its anaologues (Johdo et al. 1991a).

Compound **A** appeared very similar to α_2_-Rhodomycin II and compounds **B** and **E** to that of Rhodomycins, which perhaps could be Rhodomycin A or B depending upon whether there are two rhodosamine moieties or one. All these compounds have been previously reported for *S. purpurascens* by Brockman (1955) and Shoji (1968; Arcamone 1984).

From our study, compound **C** appeared to be similar to an ε-RMN glycoside, Epelmycin and compound **F** resembled to an γ-isoRMN glycoside, Obelmycin. To the best of our knowledge, these compounds have not yet been reported for *S. purpurascens*. They have, however been reported for mutants of *S. violaceus* (Johdo et al. 1991c), which is very closely related and a phylogenetic neighbor of *S. purpurascens* as is evident from the Figure 1.The biosynthetic pathway for rhodomycin synthesis has been elucidated by Oki (1977), according to which during the synthesis of rhodomycin (a β-RMN glycoside), ε-RMN is an intermediate. This ε-RMN can be glycosylated by a glycosyl transferase to form ε-RMN glycosides, epelmycins. It has been suggested that biosynthetic conversion from ε-RMN to β-RMN takes place at glycoside level (Johdo et al. 1991b; Johdo et al. 1991c). Therefore formation of Epelmycins which are ε-RMN glycosides can be expected to occur in *S. pupurascens* too. Similarly, Compound **D** identified as an aklavinone glycoside, Aclacinomycin A which is the main product in S. *galilaeus* has also not been reported for *S. purpurascens*. Aklavinone is the first aglycone during the synthesis of rhodomycin (Connors et al. 1990). Aklavinone can be glycosylated by a glycosyl transferase to form Aclacinomycin (Oki et al. 1979). Hence the possibility of obtaining aclacinomycins in the fermentation broth of *S. purpurascens* cannot be denied.

Compound **G** was identified as Rhodosaminyl-2-Deoxyfucosyl-α_2_ Rhodomycinone. α_2-_RMN glycosides have been designated as Alldimycins by Johdo (Johdo et al. 1991d). All Alldimycins have been reported to contain only one sugar, Rhodosamine. Our studies consistently showed the presence of 2-deoxy fucose in the chromatogram of compound **G**. This leads us to believe that compound **G** may be a new rhodomycin analogue which has not been previously reported.

Antibacterial studies showed that compound **E** which appeared similar to Rhodomycin B was the most potent compound. Its MIC was found to be 2 μg/ml which was comparable to the reported value of 0.5-1 μg/ml (Shoji et al. 1968). However, the IC_50_ value, around 15 μM (~8 μg/ml) indicating much less toxicity than the reported value of 1 μg/ml (Saito et al. 1995).

Compound **A**, identified as α_2-_Rhodomycin II and compound **F**, identified as Obelmycin were found to be less potent. The MIC of both **A** and **F** was found to be > 20 μg/ml and IC_50_ was found to be around 15 μM (~8 μg/ml), which is much higher than the reported values. Compound **F** appeared similar to Obelmycins which are γ-isoRMN glycosides. These are reported to have low bioactivity because the sugar rhodosamine is bound at C-10 of the carbon skeleton (Johdo et al. 1991e). According to Vanek et al.(1977), the position of sugar moiety on the carbon skeleton is important. Anthracycline glycosides having C-7 sugar are more active than those having it at C-10.

Thus this study resulted in the isolation of a rhodomycin producer from soil and its identification as *Streptomyces purpurascens.* The isolate was found to produce one novel Rhodomycin analogue and two known analogues being reported however, first time from *S. purpurascens*.

## Electronic supplementary material

Additional file 1: **The general structure of the aglycone moiety for most commonly used anthracyclines.** Common substituents are listed below the structure. (DOC 54 KB)

Additional file 2: Figure S1: Graphical representation of effect of carbon and nitrogen sources respectively on growth and antibiotic production of *S. purpurascens*. (A) Starch and (B) Yeast Extract appeared as best Carbon and Nitrogen sources respectively for the growth as well as for antibiotic production by *S. purpurascens*. (TIFF 789 KB)
