# Supplementary material for: Rhodomycin analogues from Streptomyces purpurascens: isolation, characterization and biological activities
Source: Springerplus. 2013 Mar 9;2:93. doi: 10.1186/2193-1801-2-93 (PMC3667366; doi:10.1186/2193-1801-2-93)
Supplement: Supplementary file 1 — Additional file 1: The general structure of the aglycone moiety for most commonly used anthracyclines. Common substituents are listed below the structure. (DOC 54 KB) [file 40064_2012_285_MOESM1_ESM.doc]

[
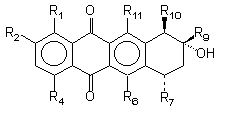
](http://users.utu.fi/jarnie/thes/fig1.gif)

Location Possible substituents

R1 H, OH, glycoside

R2 H, OH, glycoside

R4 OH, OCH_3_

R6 H, OH

R7 H, OH, glycoside

R9 CH_3_, CH_2_CH_3_, COCH_3_, COCH_2_OH, CHOHCH_3_,

CHOHCH_2_OH, CH_2_COCH_3_

R10 H, OH, COOCH_3_, glycoside

R11 H, OH
