# Supplementary figures and images for: Rhodomycin analogues from Streptomyces purpurascens: isolation, characterization and biological activities
Source: Springerplus. 2013 Mar 9;2:93. doi: 10.1186/2193-1801-2-93 (PMC3667366; doi:10.1186/2193-1801-2-93)

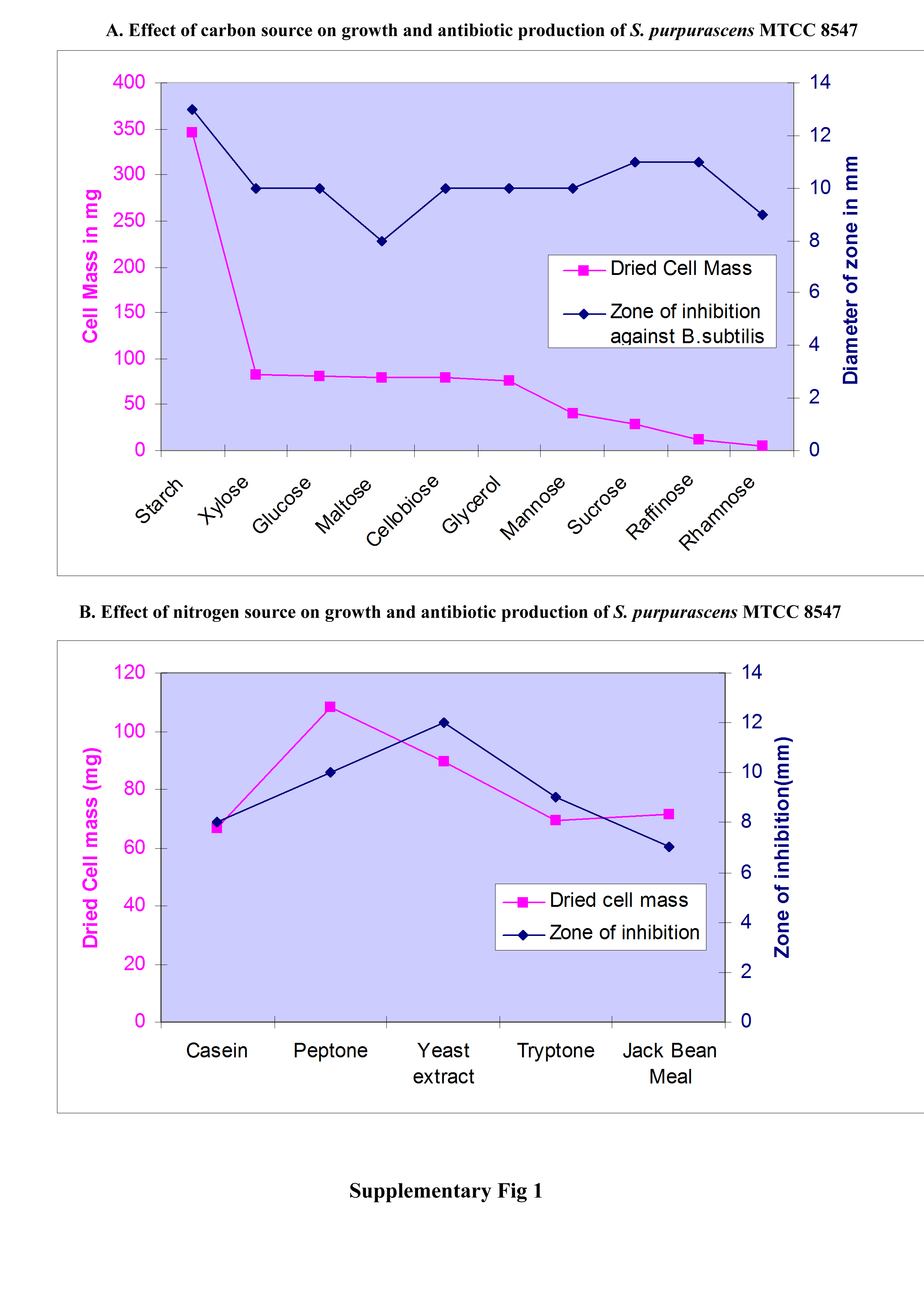

Supplement: Supplementary file 2 — Additional file 2: Figure S1: Graphical representation of effect of carbon and nitrogen sources respectively on growth and antibiotic production of S. purpurascens. (A) Starch and (B) Yeast Extract appeared as best Carbon and Nitrogen sources respectively for the growth as well as for antibiotic production by S. purpurascens. (TIFF 789 KB) [file 40064_2012_285_MOESM2_ESM.tiff]
